# The Role of Epigenetics in the Fibrotic Processes Associated with Glaucoma

**DOI:** 10.1155/2014/750459

**Published:** 2014-03-31

**Authors:** Fiona McDonnell, Colm O'Brien, Deborah Wallace

**Affiliations:** ^1^UCD School of Medicine and Medical Science, University College Dublin, Dublin, Ireland; ^2^Department of Ophthalmology, Mater Misericordiae University Hospital, Dublin, Ireland

## Abstract

Glaucoma is an optic neuropathy that affects 60 million people worldwide. The main risk factor for glaucoma is increased intraocular pressure (IOP), this is currently the only target for treatment of glaucoma. However, some patients show disease progression despite well-controlled IOP. Another possible therapeutic target is the extracellular matrix (ECM) changes in glaucoma. There is an accumulation of ECM in the lamina cribrosa (LC) and trabecular meshwork (TM) and upregulation of profibrotic factors such as transforming growth factor **β** (TGF**β**), collagen1**α**1 (COL1A1), and **α**-smooth muscle actin (**α**SMA). One method of regulating fibrosis is through epigenetics; the study of heritable changes in gene function caused by mechanisms other than changes in the underlying DNA sequence. Epigenetic mechanisms have been shown to drive renal and pulmonary fibrosis by upregulating profibrotic factors. Hypoxia alters epigenetic mechanisms through regulating the cell's response and there is a hypoxic environment in the LC and TM in glaucoma. This review looks at the role that hypoxia plays in inducing aberrant epigenetic mechanisms and the role these mechanisms play in inducing fibrosis. Evidence suggests that a hypoxic environment in glaucoma may induce aberrant epigenetic mechanisms that contribute to disease fibrosis. These may prove to be relevant therapeutic targets in glaucoma.

## 1. Introduction

Glaucoma is an optic neuropathy that affects approximately 60 million people worldwide [1]. In glaucoma, the retinal ganglion cell axons are irreversibly lost through a number of factors that combine to create the overall disease profile [2]. The factors that contribute to the disease include, but are not limited to: increased intraocular pressure (IOP), age, genetic mutations, and reduced ocular perfusion pressure (OPP) [3–7].

Within the body, there is a normal process of wound healing and scarring, however, when this process is allowed to continue unchecked, connective tissue fibrosis occurs [8, 9]. In glaucoma, fibrosis is known to occur as a build-up of extracellular matrix (ECM) materials in the trabecular meshwork (TM) at the anterior of the eye [10–12], and in the lamina cribrosa (LC) at the optic nerve head (ONH) [13–15]. This mechanism of fibrosis plays a role in the disease progression. When the TM becomes clogged with ECM, the fluid within the eye, the aqueous humor (AH) cannot easily exit via its normal pathway and the pressure within the eye subsequently increases. This increase in intraocular pressure (IOP) is one of the main risk factors associated with the progression of glaucoma [4, 16] and is currently the only target for treatment in clinical use [17]. Following the increased IOP, structural damage (cupping) occurs at the optic nerve head which is associated with the loss of retinal ganglion cells (RGC) and the loss of vision seen in glaucoma [18, 19]. The lamina cribrosa is a fenestrated region of the ONH through which the nerves travel to the brain [2]. In glaucoma, there is backward bowing of the LC, and this likely puts pressure on the optic nerves, compressing them, which then leads to loss of vision [2, 20]. This damage at the ONH region is associated with an accumulation of ECM molecules at the lamina cribrosa [4, 14, 15, 21, 22].

There are a number of profibrotic factors that have been found to have increased levels in the AH and TM of glaucomatous eyes. These include the cytokine transforming growth factor *β* (TGF*β*) [23], the matricellular proteins thrombospondin-1 (TSP1) [24], and connective tissue growth factor (CTGF) [25]. The roles of TGF*β* and CTGF in fibrosis are well established [26–40]; TGF*β* acts through Smad proteins to activate ECM proteins such as collagen and PAI-1 to drive fibrosis [41–44]. TGF*β* binds to two different serine threonine kinase receptors—Type I and Type II. The Type II receptor is constitutively active and when a ligand binds to this receptor, it complexes with and phosphorylates the Type I receptor—this then phosphorylates Smad proteins which translocate to the nucleus to regulate gene transcription [45]. TGF*β* is a known regulator of CTGF which acts as a downstream mediator for TGF*β* [26, 27, 32, 46]; however, this mechanism is poorly understood.

These factors have been shown to be involved in ECM production [35, 47, 48], and as CTGF and TGF*β* are present in the AH of human eyes [23, 25], it is possible that they drive the production of ECM in the TM and at the LC. As previous work from our group has shown, there are increased levels of both TGF*β*1 and TSP-1 in the LC cells of glaucomatous eyes [49] and increased levels of CTGF in the AH of glaucomatous eyes affecting the TM [25]. Further, it has been shown in a number of fibrotic diseases that TGF*β* plays a role in mediating fibrosis and causes an increase in ECM deposition [37, 50, 51]. Studies show that the same is true in the process of glaucoma—increased levels of TGF*β* lead to increased ECM deposition in the TM and LC of glaucomatous eyes [51].

In an attempt to combat fibrosis, a number of therapeutic approaches have been studied. Baricos et al. showed that TGF*β*1 inhibited ECM degradation, and blocking TGF*β*1 using an anti-TGF*β*1 antibody increased the degradation of ECM in human mesangial cells (HMCs) [34]. Further, a study of glomerulosclerosis in rat models demonstrated that an anti-TGF*β* antibody significantly reduced fibrosis. This study demonstrated decreased mRNA expression of TGF*β* isoforms and collagen type III in the presence of the antibody and showed a reduction in the level of fibrosis and sclerosis seen in the kidney [52]. It was shown that transfecting TM cells with small interfering RNA (siRNA) for CTGF inhibited TGF*β*2 induced upregulation of CTGF and fibronectin [47]. A recombinant monoclonal neutralizing antibody (mAb) to human TGF*β*2 was shown to significantly improve the outcome of glaucoma filtration surgery in a rabbit model of conjunctival scarring [53]. Work by our lab has shown that a humanized monoclonal anti-CTGF antibody FG-3019 was able to effectively block ECM production in LC and TM cells treated with AH samples from pseudoexfoliation glaucoma (PXFG), primary open angle glaucoma (POAG), and hydrogen peroxide, as shown by a significant reduction in the expression of profibrotic genes [54].

TSP1 has been shown to induce the active form of TGF*β* by inducing its dissociation from a protein that binds to and keeps it, in its latent, inactive form, thereby regulating its pathway [55]. This can result in the induction of a fibrotic phenotype through modulation of TGF*β* activity [55]. A study in mice demonstrated that knocking out TSP1 significantly lowered IOP compared to wild type mice and that this may be due to altered ECM and aqueous humour outflow in the knockout mice [56].

**Figure 1:**
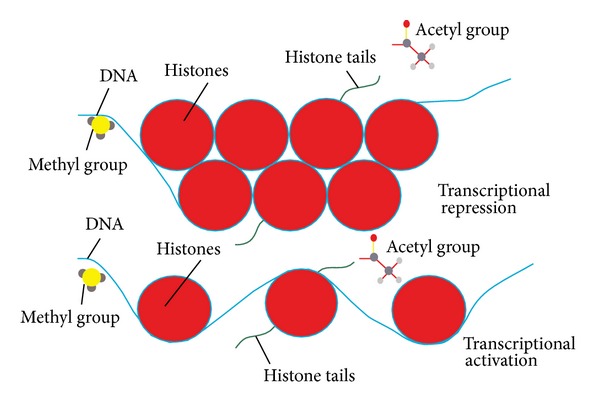
Epigenetic mechanisms. DNA is wrapped around proteins called histones; this forms the core DNA package called the nucleosome. When the DNA is tightly wrapped, transcription factors cannot bind and transcription is repressed. When the DNA is more loosely wrapped, transcription is more active as transcription factors can bind. DNA methylation is when a methyl group is added to the DNA strand and is associated with transcriptional repression [58]. Histone acetylation is the addition of acetyl groups to the histone tails and this is associated with transcriptional activation. Histone deacetylation is the removal of acetyl groups which is associated with transcriptional repression [59].

However, there is another method by which fibrosis may be regulated, and this is through epigenetics. Epigenetics is the study of heritable changes in gene function caused by mechanisms other than changes in the underlying DNA sequence [57]. It involves DNA methylation [58] and histone modifications including acetylation/deacetylation and methylation [59]. It has been proposed that these epigenetic processes play a role in the progression of fibrosis in a number of diseases [60–62] (Figure 1). Previously, it has been shown that epigenetic mechanisms can influence the activity of TSP1 in cancers [63, 64], so it is likely that there may be a similar effect in fibrotic disease. Further, senescent myofibroblast resistance to apoptosis has been linked to both global and locus-specific histone modifications like methylation and acetylation [65]. Micro-RNAs (miRNAs) have been established as regulators of fibrosis in cardiac, kidney, and lung fibrosis [66–68]. It has recently been demonstrated that epigenetic mechanisms may play a role in the regulation of miRNAs and that miRNAs use epigenetic mechanisms to mediate their downstream effects in cardiovascular disease and pulmonary fibrosis [69–71].

There is evidence of a hypoxic environment in glaucomatous eyes, both in the AH and at the ONH. Oxidative stress markers have been found in the AH of glaucomatous eyes [72] and a study by our laboratory showed that LC cells from glaucomatous donors show increased markers of oxidative stress (increased production of reactive oxygen species (ROS)) and compromised antioxidant activity [73]. A hypoxic environment has also been shown in studies that demonstrated the presence of hypoxia-inducible factor 1*α* (HIF1*α*) in the ONH, which is an indicator of hypoxia [74]. Hypoxia has been shown to induce an epigenetic response which regulates the cellular response to the hypoxic insult [75]. The induction of aberrant epigenetic modifications could potentially be through the hypoxic environment caused by the oxidative stress present in glaucomatous eyes.

**Figure 2:**
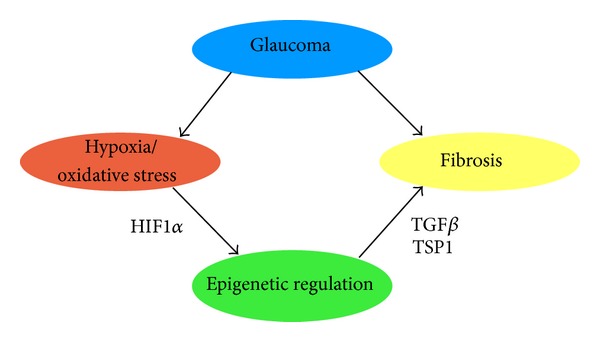
The potential role of hypoxia and epigenetics in the fibrosis seen in glaucoma. The hypoxic environment in glaucoma [74] may cause the epigenetic profile of the cells to change bringing about a more fibrotic phenotype [61, 75].

TGF*β*, TSP-1, and a hypoxic environment can contribute to the disease pathogenesis of glaucoma and as there is a role for epigenetics in each of these, we will look at the epigenetic mechanisms that may be a part of the overall process (Figure 2).

## 2. TGF***β***

### 2.1. TGF*β* and Fibrosis

The TGF*β* family of cytokines contains a number of multifunctional proteins that are involved in the regulation of a variety of gene products and cellular processes [13, 48, 87–89]. There are three isoforms of TGF*β* (1, 2, and 3) and each of these are encoded by a different gene [90]. Members of this family are involved in inflammation, wound healing, and ECM production and accumulation, among others [38, 90]. TGF*β*1 and TGF*β*2 have been shown to be the predominant isoforms in the eye; in the ONH and the AH [91, 92]. TGF*β*1 has been found to be elevated in PXFG, and it plays a significant role in extracellular matrix formation and accumulation in PEX syndrome [23, 93]. TGF*β*2 levels have been found to have been increased in POAG [92].

The TM plays an integral role in the outflow pathway through which the aqueous humor leaves the eye. In glaucoma, the TM becomes clogged with ECM molecules [10–12] preventing the AH from being drained and creating an increase in intraocular pressure. Studies demonstrated that TGF*β* acts through a number of pathways in the TM which leads to increased ECM deposition. Using exogenous TGF*β*, it has been shown that the process takes place in 4 mechanisms; it increases the synthesis of ECM molecules in TM cells [51]; it increases the expression of plasminogen activator inhibitor (PAI)-1 which prevents the activation of matrix metalloproteinases (MMPs) that play a role in degrading ECM [51]; it increases the transglutaminase-mediated irreversible cross-linking of ECM components by TM cells [94]; and it inhibits the proliferation of TM cells [95]. Treatment of TM cells with TGF*β*2 stimulates the expression of ECM genes including collagens, fibrillin, laminin, and elastin. TGF*β*2 treatment can also increase the expression of fibronectin and PAI-1 [38, 51].

There is an increase in TGF*β*2 levels in the ONH region of the eye; this is mostly localised to the astrocytes present in the nerve bundles in the LC [96]. TGF*β* is also able to induce TSP1 which in turn activates TGF*β* [24]. In the ONH, the ECM usually provides a frame and resilience for the nerves [97], however, in glaucoma, the ECM is altered by basement membrane thickening along the lamina cribrosa beams and also changes in collagen and elastin fibres within the beams [21]. TGF*β*2 induces the synthesis of collagens and fibronectin [38, 51], all of which could contribute to the thickening of the basement membrane. It has been shown that TM cells secrete endogenous TGF*β*1 [98], and it has also been demonstrated that exogenous TGF*β* increases the synthesis and deposition of ECM proteins in LC cells, namely, fibronectin, collagens, elastin, and PAI-1 [96], so it is likely that endogenous TGF*β* has a similar effect in these cells. Therefore, an increase in the amount of endogenous TGF*β* secreted from the cells could increase ECM deposition at the TM. Work from our lab has shown that TGF*β*1 is increased in glaucomatous LC cells in comparison to normal LC cells [49] and treatment of normal LC cells with exogenous TGF*β*1 upregulated profibrotic genes such as CTGF, collagen I, and thrombospondin [99]. The use of glaucomatous-like stimulus (cyclical mechanical strain—cell stretch and a hypoxic environment—1% O_2_) upregulated genes associated with the ECM, such as CTGF, Collagen I, Elastin, TSP-1 macrophage migration inhibitory factor 1 (MIF), discoidin domain receptor family member 1 (DDR1/TrkE), and Insulin-like growth factor 2 receptor (IGFR2) seen in the LC region of glaucoma [84, 100].

There are a number of anti-TGF*β* therapies in research and clinical trials; one such therapy is SB-431542. This is an inhibitor of the TGF*β* Type I Receptor kinase activity and so an inhibitor of the TGF*β* pathway. A study on the inhibition of TGF*β*1-induced ECM using this therapy demonstrated that SB-431542 decreased TGF*β*1-induced upregulation of fibronectin and collagen1a1 in a renal epithelial carcinoma cell line, both of which are ECM proteins [101]. A second study by Mori et al. similarly showed that SB-431542 prevented the TGF*β*-induced stimulation of collagen, fibronectin, and CTGF in skin fibroblasts [102]. The TGF*β*2 antibody CAT-152 has been shown to reduce collagen deposition in the subconjunctival following subconjunctival glaucoma surgery and improved surgical outcome in rabbits [103].

### 2.2. Epigenetic Control of TGF*β* Expression

**Figure 3:**
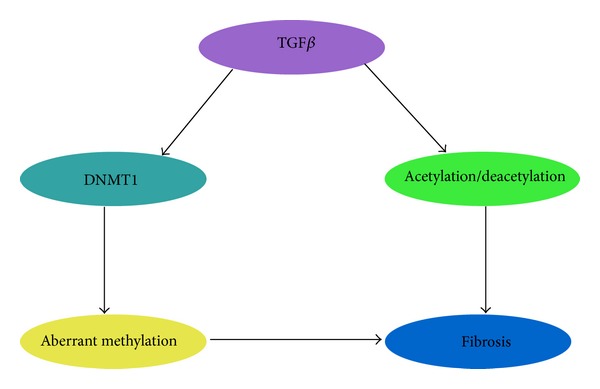
Epigenetic regulation of the TGF*β* pathway. TGF*β* has been shown to upregulate the expression of DNMT1 which causes aberrant methylation [76] leading to a more fibrotic phenotype. Furthermore, TGF*β* has been shown to decrease acetylation in corneal fibroblasts causing them to remain active and leading to fibrosis [77]. In contrast to its role in corneal fibroblasts, TGF*β* enhances Smad 2/3 acetylation leading to increased activity of these Smads [78].

TGF*β* has also been demonstrated to be regulated through epigenetic processes including DNA methylation and histone acetylation/deacetylation and methylation [76, 77, 104] (Figure 3). Alterations in the histone status of promoters of target genes may lead to a difference in the TGF*β*-mediated transcription profile, and so they may determine the cell's response to TGF*β*. A number of studies have shown that altered histone modifications can affect TGF*β* functions within the cell [77]. The acetylation/deacetylation of histones can determine how TGF*β* is able to induce a cell response to stimuli [105]. This was demonstrated in the case of corneal fibroblasts, in the normal process of wound healing, these cells transition from a proinflammatory to a profibrotic phenotype. The acetylation status of corneal fibroblasts was examined and it was found that TGF*β* partially inhibited histone acetylation. This was reversed by a histone deacetylase inhibitor, trichostatin A (TSA), which also reversed TGF*β*-induced upregulation of profibrotic factors. Therefore, the modification of histone acetylation in corneal fibroblasts was involved in TGF*β* regulation of cell transition to a profibrotic state [77]. A study by the same authors showed that HDAC inhibitors TSA and sodium butyrate (NaBu) blocked the TGF*β*-induced upregulation of *α*SMA and collagen I in corneal stromal cells [106]. Conversely, Inoue et al. demonstrated that Smad-2 and -3 acetylation by the histone acetyltransferases CBP/P300 were enhanced by TGF*β* in renal and liver cell lines [78]. This demonstrates that TGF*β* plays a diverse role in the epigenetic regulation of fibrosis by both histone acetylation and histone deacetylation to drive fibrosis.

A further example of how histone acetylation/deacetylation can regulate TGF*β* activity was seen in corneal fibroblasts treated with TSA demonstrated inhibition of TGF*β*-induced reactive oxygen species (ROS) accumulation and myofibroblast differentiation [104].

Bruna et al. showed that TGF*β* induces proliferation in one cell line (U373MG) of glioblastoma cells, but inhibits it in another (U87MG) and this is connected to TGF*β* induced platelet-derived growth factor B (PDGFB). This result can be explained by the methylation status of PDGFB in the two cell lines. In one of the cell lines (U87MG), the PDGFB promoter was methylated, which blocked TGF*β*-Smad signalling in this cell line. However, in the other cell line (U373MG), the promoter was not epigenetically suppressed and so TGF*β*-Smad signalling was active. Therefore, the DNA methylation status of the cells is able to determine if the cell response is controlled by TGF*β* activity [89]. Furthermore, the TGF*β* signalling pathway has been shown to be suppressed through methylation. A number of genes were analysed and were shown to be methylated; these genes include TGF*β* receptor 2 (TGF*β*R2) and TSP-1. Treating the cells with DNA methyltransferase (DNMT) (responsible for the transfer of methyl groups to the DNA) inhibitors increased the TGF*β* pathway activity [76].

## 3. Thrombospondin

### 3.1. Thrombospondin-1 and TGF*β*

Thrombospondin-1 is a matricellular, multifunctional protein that is expressed by cell types involved in wound healing. It is known to regulate cellular events in tissue repair, including cell adhesion [107], apoptosis [108], ECM expression, and organization through modulation of growth factors [109–111]. TSP1 has been shown, by our lab, to be increased in glaucomatous LC cells compared to normal LC cells [49], and it has also been demonstrated that TSP1 is increased in glaucomatous TM cells [24]. It has been shown to be expressed at increased levels in renal tissues undergoing fibrosis [110]. In a model of renal fibrosis, it was found that TSP1 is an important mediator of the disease, and its knockout reduced renal inflammation and fibrosis [112].

TGF*β* is secreted from cells in a latent form which associates with the latency-associated peptide (LAP) [113]. Dissociation of TGF*β* from LAP can be induced by TSP1, and this step is necessary for the activation of TGF*β* [109]. TGF*β* activation by TSP1 is achieved through a conformational change and is required for the regulation of the TGF*β* signalling pathway [114]. TSP1-TGF*β* binding does not affect TGF*β* activity; this is possibly because TSP1 may play a role in aiding TGF*β* presentation to cell surface receptors [115].

Studies have shown that TSP1 activates TGF*β* secreted from a number of cell types; endothelial cells, mesangial cells, and cardiac fibroblasts [111, 116, 117]. It has also been demonstrated that TSP1 activates TGF*β* in fibrotic disease [118]. While activation of TGF*β* by TSP1 is necessary during development to give a normal phenotype [55], it is very likely that the main role for TSP1 in regulating TGF*β* activation is during the processes of injury, stress, and in pathological conditions. In glaucoma, it is likely that TSP1 is necessary for TGF*β* activity, as it is present in high levels in glaucomatous TM [24]. Further, a number of studies have demonstrated that treating TM cells with glaucomatous-like stimuli (TGF*β*, cyclical stretch) increases TSP1 in these cells [51, 99, 100]. Interestingly, it has been demonstrated that knocking out TSP1 in mice results in a significantly lower IOP when compared to wild type [56]. This reduction in IOP was attributed to a change in the ECM of these mice and to an increase in the rate of aqueous turnover. These data suggest that TSP1 plays a role in both glaucoma and in the regulation of ECM.

### 3.2. TSP1 and Epigenetics

There are also methods by which TSP1 itself is regulated in the disease context. Aberrant TSP1 methylation has been seen to effect TSP1 regulation of TGF*β* in some cancers. In colorectal cancer, TGF*β* inhibits cell proliferation and induces apoptosis of epithelial calls. In this form of cancer, TGF*β* acts as a tumour suppressing pathway in the initial disease stages [63]. As previously discussed, TSP1 regulates the TGF*β* pathway by activating TGF*β* [119]. In colorectal cancer, TSP1 has been found to be aberrantly methylated, and it is thought that it may promote tumorigenesis [63]. As it is known that the TGF*β* pathway is regulated by TSP1, it has now been hypothesised that TSP1 promotes the formation of tumours through inhibiting the TGF*β* pathway when its methylation status is altered. For example, Rojas et al. demonstrated that the hypermethylation of the TSP1 promoter in colorectal cancer suppressed TSP1 mRNA and protein expression. The reduced levels of TSP1 inhibited the activation of TGF*β* in the disease and so suppressed the TGF*β* signalling pathways which are beneficial in the first stages of disease. Another study of gastric cardia adenocarcinoma demonstrated that while TSP1 promoter methylation affected the mRNA and protein levels of thrombospondin 1, there were no significant effects on TGF*β* expression, although a nonsignificant decrease of active TGF*β* was seen in patients with TSP1 hypermethylation [64] indicating that the methylation of TSP1 causes downregulation of TSP1 and therefore decreased active TGF*β*.

## 4. Hypoxia

### 4.1. Hypoxia and Glaucoma

**Figure 4:**
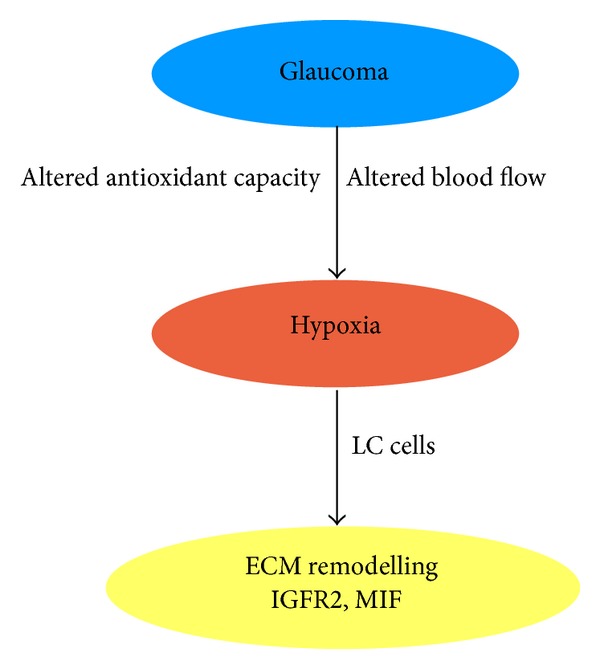
Hypoxia induces the expression of ECM remodelling genes in LC cells. It has been hypothesized that the increased IOP in glaucoma causes the blood flow to the eye to be altered, and this may be a cause of the hypoxic environment seen in glaucomatous eyes [79–83]. In addition to the altered blood flow, our group has shown that glaucomatous LC cells have decreased antioxidant capacity [73]. We have also shown that hypoxia can induce increased expression of ECM remodelling genes such as insulin-like growth factor 2 receptor (IGFR2) and macrophage migration inhibitory factor 1 (MIF) in LC cells [84].

Hypoxia is a state in which there is not enough oxygen entering the tissues for them to function as normal. It is linked to age as a natural feature in many organs [120]. However the cellular response to hypoxia may result in the increased expression of survival factors [75]. There is evidence that there is a hypoxic environment present in glaucoma [74], and that this hypoxic state induces retinal ganglion cell (RGC) death which is part of the disease pathogenesis [121]. Our lab has also shown evidence of oxidative stress in glaucomatous LC cells and a decreased capacity of the cells to counteract the oxidative stress [73]. It has been shown that ocular blood flow is reduced in patients with glaucoma [79–81, 122, 123], and specifically in those in which the disease is progressing. A decrease in blood flow could lead to a decreased level of oxygen—giving a hypoxic state [82]. Increased IOP and decreased ocular perfusion pressure [4–6, 81, 83] can both effect the ocular blood flow, and so the hypoxic environment and the oxidative stress may be a result of increased IOP seen in glaucoma [82, 124]. Following from this, it has been found that a fluctuating blood flow is likely to be a cause of glaucomatous damage. Further, work from our laboratory demonstrated that in vitro hypoxia can cause LC cells to produce genes involved in ECM production and remodelling including insulin-like growth factor 2 receptor (IGFR2) and macrophage migration inhibitory factor 1 (MIF) (Figure 4). The HIF families are the key regulators of the cell's adaptive response to hypoxia, and they control the expression of many genes involved in many cell processes, including fibrosis [125, 126]. HIF1*α* regulates gene expression through hypoxia response elements (HRE) present in the promoter regions of target genes. Hung et al. identified a HRE in the TGF*β*1 promoter; this likely allows it to be regulated by hypoxia [86].

It is believed that RGC death may be caused by a hypoxia-induced apoptotic pathway [74, 121]. Hypoxic states induce the expression of HIF1*α*, which is responsible for the transcriptional responses that allow cells to adapt to a hypoxic environment [75]. Tezel and Wax conducted a study where they found evidence of increased HIF1*α* expression in the ONH of glaucomatous eyes [74]. Regions of HIF1*α* expression indicate areas of decreased oxygen and so hypoxic stress, which suggests that there is tissue hypoxia in glaucoma and that this plays a role in the disease. Also, areas of HIF1*α* expression were also found to correlate to areas of visual field defects in patients [74].

### 4.2. Hypoxia and Fibrosis

A role for hypoxia in fibrosis has been suggested in different experimental models such as adult wound repair [127] and cirrhosis [128]. It has been shown that hypoxia up-regulates collagenase IV expression in cardiomyocytes [129], and increases interstitial collagen in renal tubulointerstitial cells, while decreasing collagen IV expression [130]. A study by Corpechot et al. demonstrated that hypoxia in hepatic stellate cells may directly affect the quantitative and qualitative change in the ECM during liver fibrogenesis [128]. As mentioned before, collagens are ECM proteins and so the upregulation of collagens leads to an increase in ECM build-up. Along with this, it has also been demonstrated that hypoxia can mediate the induction of TGF*β* mRNA in a hepatoma cell line [131] and in dermal fibroblasts [132]. It has been hypothesised that hypoxia may act in converting TGF*β* from its latent form to its active form [133] as there is a HRE in the TGF*β*1 promoter [86], which allows it to be regulated by hypoxia.

In tubulointerstitial cells, it is believed that altered microvasculature brings about a hypoxic environment that causes a fibrotic response “the chronic hypoxia hypothesis” [134]. As mentioned before, there is altered blood flow in glaucoma [82], and this may be a similar mechanism to that seen in the tubulointerstitial cells. In tubular epithelial cells, hypoxia has been seen to induce changes in expression of genes that play a role in cell adaptation to stimuli [135]. Hypoxia has been shown to induce the expression of fibrogenic factors including TGF*β* and angiogenic factors including vascular endothelial growth factor (VEGF) [136]. The cell's response to hypoxia acts with other fibrogenic stimuli to add to the fibro-vascular response, as well as inducing its own changes [137].

Hypoxia has also been shown to promote a more fibrotic phenotype in fibroblasts, which are ECM-producing cells [137]. It does this by increasing proliferation, and enhancing cell differentiation and contraction. It also alters the metabolism of the ECM and upregulates proteins associated with matrix production [138] and acts to decrease expression of proteins that degrade the ECM, such as matrix metalloproteinases [133]. There is evidence that hypoxia may also be involved in suppressing the apoptosis of fibroblasts [139]. A study by Zhang et al. showed that hypoxia increases the TSP1 pathway that activates TGF*β* signalling in human umbilical vein endothelial cells [140]—suggesting that hypoxia may affect the expression of TGF*β* through a number of regulatory processes. It was demonstrated that HIF1*α* could induce the expression of TSP1 in cells grown in hypoxic conditions. This was achieved through HRE binding near the transcription starting site, as HIF-1*α* was demonstrated to bind to this site in a hypoxic environment [141]. HIF1*α* has been shown to be the most active in regulating gene expression associated with the hypoxic response [136]. It may play a role in the promotion of fibrosis through the induction of epithelial-to-mesenchymal transition (EMT) in which cells change from an epithelial phenotype to a more fibrotic, myofibroblast phenotype [136].

### 4.3. Hypoxia and Epigenetics

**Figure 5:**
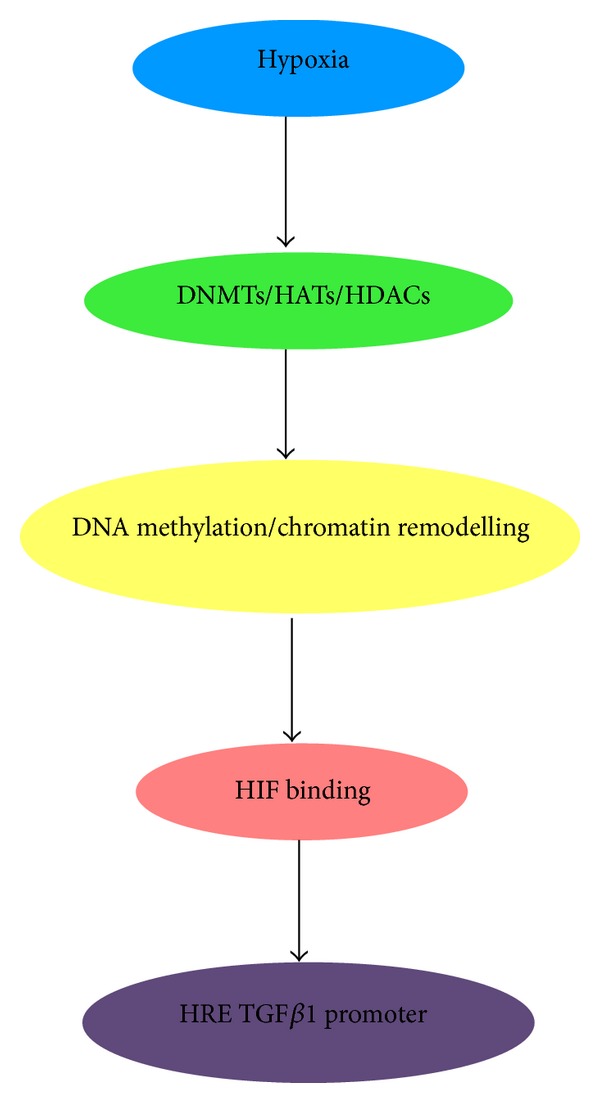
Epigenetic changes allow HIF binding to HIF response elements (HREs). A hypoxic environment within a cell can cause normal epigenetic mechanisms to be changed, altering the level of DNA methylation and/or modifying chromatin conformation allowing HIF1*α* to bind to the HRE on the gene [85]. The TGF*β*1 promoter has been previously shown to contain a HRE [86], and so hypoxia can alter TGF*β*1 expression through HIF1*α*.

A study by Watson et al. showed that chronic hypoxia in prostate cells induced an alteration in DNA methylation and histone acetylation. In the absence of HIF1*α* which is responsible for downstream mediation of the hypoxic phenotype, epigenetic alterations may take over this role in establishing and maintaining the hypoxia related phenotype [75]. Furthermore, it has been suggested that HIF may require epigenetic mechanisms to aid in the initiation and maintenance of the cell phenotype in a hypoxic environment. In the presence of oxygen, HIF1*α* is regulated through hydroxylation, ubiquitination, and degradation by prolyl hydroxylase enzymes (PHD) [142]. In the absence of oxygen, this is inhibited which allows for HIF1*α* stabilisation and activation [143]. HIF1*α* regulates gene expression through hypoxia response elements (HRE) present in the promoter regions of target genes [144]. This binding can be affected through DNA methylation and histone modification, which may maintain a favourable chromatin conformation around HRE sites (Figure 5).

The creb binding protein/p300 coactivator (CBP/P300) is a histone acetyltransferase (HAT) that functions by adding acetyl groups to histones and driving gene transcription. It is known to associate with HIF1*α* to coactivate hypoxia-inducible genes [145]. This association can be affected by histone deacetylases (HDACs) and also by HDAC inhibitors. HDAC3 is a binding partner of HIF1*α* that aids in the regulation of its stability during the hypoxic response [146]. HIF1*α* binding may also be affected by the methylation of CpG sites at the HRE. DNA hypomethylation, which is typically associated with more active gene transcription, has been shown to be induced by tumour hypoxia [147].

Additionally, there is also evidence to suggest that epigenetic modifications induced by hypoxia play a role independent of HIF. Modified histones in this case can directly interact with promoter regions of hypoxia-inducible genes [147, 148]. Robinson et al. showed that DNA hypermethylation induced by hypoxia in human pulmonary fibroblasts is associated with the development of a profibrotic phenotype. Thy-1 is a glycoprotein that can affect intracellular signalling pathways. Its absence on fibroblasts is associated with a myofibroblast phenotype. This group showed that hypermethylation of the Thy-1 promoter was induced by hypoxia and led to a myofibroblast phenotype and treatment with 5-aza-2-deoxycytidine caused an increase in Thy-1 expression [61].

## 5. Epigenetics in Ocular Diseases

There are a number of studies that have suggested a role for epigenetics in ophthalmology. A study of monozygotic and dizygotic twins with discordant age-related macular degeneration (AMD) showed differential methylation of the promoter regions of 231 genes [149]. A further study of AMD conducted bisulfite sequencing of the retinal pigment epithelium (RPE); there was hypermethylation of the promoter regions of two glutathione S transferase (GTSM) isoforms, which resulted in decreased mRNA and protein [150]. Importantly, GTSMs are involved in defending against ROS, which have been shown by our lab to be upregulated in glaucomatous cells [73]. The hypermethylation of the *α*A-crystallin (CRYAA) promoter also coincided with downregulation of mRNA and protein in age-related cataract [151]. Zhou et al. showed that methylation of the collagen 1a1 (COL1A1) promoter could play a role in the development of myopia [152]. In a mouse model of optic nerve crush (ONC), there was an increase in the nuclear localisation and activity of HDACs 2 and 3 and a corresponding increase in histone 4 deacetylation; this was associated with RGC death [153]. Further, the silencing of the Fem1cR gene by histone deacetylation has been connected to RGC death in a DBA/2J mouse model of glaucoma [154]. In addition to this, there is a role for epigenetics in diabetic retinopathy; it has been shown that in streptozotocin (STZ) treated rats kept under poor glycemic control, there is increased HDACs 1, 2, and 8 in the retina and retinal endothelial cells [155].

## 6. Future Perspectives on Epigenetic Therapies in Glaucoma

Currently there are a number of epigenetic treatments being used to treat myelodysplastic syndromes and cutaneous T-Cell lymphoma. DNMT inhibitors Azacitidine and Decitabine are used to treat myelodysplastic syndromes [156]. These DNMT inhibitors cause DNA hypomethylation which has resulted in an improved survival rate for patients with myelodysplastic syndromes. These drugs were well tolerated in patients during clinical trials. This indicates that these agents may be good candidates for glaucoma therapies, if it can be demonstrated that there is aberrant DNA methylation occurring in glaucoma.

HDAC inhibitors such as Vorinostat and Romidepsin are in use to treat cutaneous T-Cell lymphoma [157]. These drugs are used when a patient relapses and other treatments are not effective. Vorinostat is a pan-HDAC inhibitor, and so inhibits class I, II, and IV HDACs. There are some minor side-effects associated with this drug, although it is overall well-tolerated. Romidepsin is similarly a pan-HDAC inhibitor also targeting class I, II, and IV HDACs. Some minor side effects were found in clinical trials but the treatments proved very effective. Similarly, if it can be shown that the mechanisms of histone acetylation/deacetylation are altered in glaucoma, these treatments may be an option for glaucoma. Further, it has been demonstrated that HDAC inhibitors may alter DNA methylation levels [158–161]. Sanders et al. found that treating rat lung fibroblasts with TSA demethylated previously hypermethylated sites of the Thy-1 promoter region [161]; TSA also upregulated methyltransferase activity in these cells [161]. Another study demonstrated that TSA reduced the global DNA methylation of cancer cell lines and downregulated the DNMT1 protein and also altered DNMT1 activity [160]. This demonstrates that there may be a synergistic role for these epigenetic mechanisms, and so combination therapies may be beneficial in treating aberrant epigenetic mechanisms in fibrotic diseases.

As there are already epigenetic treatments in clinical use, research into the role of epigenetics in glaucoma may offer potential new avenues of therapy to treat the disease. Furthermore, as previously mentioned, increased IOP is the only target for treatment, and discovering more about the role of epigenetics may provide a target for the underlying causes of the disease.

## 7. Conclusion

Glaucoma is a multifactorial disease in which all of the above elements play a role. What we can take from the current information is that there is still much to be discovered about the different aspects of the disease pathogenesis. Of particular importance is the role of epigenetics within some of the contributing factors to the disease and how the overall epigenetic profile of a glaucomatous eye differs from that of a normal eye. When taking into account the roles of TGF*β* and TSP1 in glaucoma, it is clear that these have an epigenetic aspect which contributes to the activity of these proteins in a number of diseases, and so it may play a part in how TGF*β* and TSP1 control the cellular response in the glaucomatous environment.

This also links to the role that hypoxia plays in regulating the epigenetic profile of glaucoma, as hypoxia also plays a role in the regulation of TGF*β* and TSP1. The hypoxic and oxidative stress environment found in glaucoma is thought to play a significant part in the disease pathogenesis through HIF1*α* and the induction of aberrant epigenetic modification. Epigenetic alterations allow a cell to adapt to the hypoxic environment and therefore change its phenotype—possibly to a profibrotic one in the context of glaucoma.
